# Profiles of immune cell infiltration and immune-related genes in the tumor microenvironment of osteosarcoma

**DOI:** 10.18632/aging.102824

**Published:** 2020-02-09

**Authors:** Chi Zhang, Jing-Hui Zheng, Zong-Han Lin, Hao-Yuan Lv, Zhuo-Miao Ye, Yue-Ping Chen, Xiao-Yun Zhang

**Affiliations:** 1 Graduate School, Guangxi University of Chinese Medicine, Nanning 530001, China; 2 Department of Cardiology, Ruikang Hospital Affiliated to Guangxi University of Chinese Medicine, Nanning 530011, China; 3 Department of Orthopedics, Ruikang Hospital Affiliated to Guangxi University of Chinese Medicine, Nanning 530011, China; 4 Department of Orthopedics, Hubei University of Chinese Medicine Huangjiahu Hospital, Wuhan 430065, China; 5 Ruikang School of Clinical Medicine, Guangxi University of Chinese Medicine, Nanning 530001, China

**Keywords:** bioinformatics analysis, TCGA, gene set enrichment analysis, prognosis, biomarker

## Abstract

This work aimed to investigate tumor-infiltrating immune cells (TIICs) and immune-associated genes in the tumor microenvironment of osteosarcoma. An algorithm known as ESTIMATE was applied for immune score assessment, and osteosarcoma cases were assigned to the high and low immune score groups. Immune-associated genes between these groups were compared, and an optimal immune-related risk model was built by Cox regression analyses. The deconvolution algorithm (referred to as CIBERSORT) was applied to assess 22 TIICs for their amounts in the osteosarcoma microenvironment. Osteosarcoma cases with high immune score had significantly improved outcome (P<0.01). The proportions of naive B cells and M0 macrophages were significantly lower in high immune score tissues compared with the low immune score group (P<0.05), while the amounts of M1 macrophages, M2 macrophages, and resting dendritic cells were significantly higher (P<0.05). Important immune-associated genes were determined to generate a prognostic model by Cox regression analysis. Interestingly, cases with high risk score had poor outcome (P<0.01). The areas under the curve (AUC) for the risk model in predicting 1, 3 and 5-year survival were 0.634, 0.781, and 0.809, respectively. Gene set enrichment analysis suggested immunosuppression in high-risk osteosarcoma patients, in association with poor outcome.

## INTRODUCTION

Osteosarcoma (OS), a common primary bone malignancy, tends to metastasize [1]. Between 1973 and 2012, the overall incidence rate of OS was 4.5 per million in the United States [2]. According to statistics reported in the United States from 2007 to 2013, the five-year relative survival rates of OS patients were 69.8% and 65.5% in the ages from birth to 14 years and 15 to 19 years, respectively [3]. Currently, new OS cases are administered neoadjuvant chemotherapy and surgery to remove the primary and overt metastatic tumors, with postoperative adjuvant chemotherapy; this has resulted in increased overall survival in OS [4]. However, drug resistance has worsened patient prognosis. Therefore, it is important to develop additional efficient therapeutics to improve survival in OS.

As an emerging treatment, immunotherapy has shown promising results for some cancers, including hepatocellular carcinoma and breast cancer [5, 6]. The tumor microenvironment (TME), a mixture that consists of mesenchymal cells, tumor-infiltrating immune cells (TIICs), endothelial cells, extracellular matrix molecules and inflammatory mediators [7], provides all metabolites and factors for controlling proliferation, dissemination, dormancy, and drug resistance in OS cells [8]. It was suggested that the TME plays a critical role in OS development [9]. In the TME, TIICs constitute the major type of non-tumor components reported to be valuable for prognostic assessment in OS [10]. Thus, improving immunotherapy efficacy in OS by systematically assessing the TME’s immune properties and determining TIIC distribution and functions is of prime importance.

An algorithm has been developed to predict the levels of TIICs using gene expression data from the cancer genome atlas (TCGA) (https://portal.gdc.cancer.gov/), and immune score could be calculated for predicting immune cell infiltration, by analyzing a specific gene expression signature of TIICs [11]. Recently, several studies have applied this algorithm to glioblastoma multiforme [12] and clear cell renal cell carcinoma [13], showing the feasibility of such big-data based algorithms, although the immune scores of OS cases from the TCGA database have not been investigated in detail. Moreover, Cell type Identification By Estimating Relative Subsets Of RNA Transcripts (CIBERSORT), can use the deconvolution technique to assess the levels of 22 TIICs in large amounts of heterogeneous samples [14]. CIBERSORT has been successfully applied for identifying TIIC landscapes and their associations with prognosis in colorectal, gastric and breast cancer [15–17].

To increase immunotherapy efficacy, determining immune-associated prognostic biomarkers is especially pivotal. Here, we calculated immune score of OS cohorts in the TCGA database by taking advantage of the algorithm, known as ESTIMATE, retrieved immune-associated differentially expressed genes (DEGs) in OS, and built a predictive risk model to estimate patient outcome. Importantly, we also evaluated the associations of the immune-related risk score with the levels of TIICs and immune pathways.

## RESULTS

### The immune score is tightly associated with overall survival in OS

**Figure 1:**
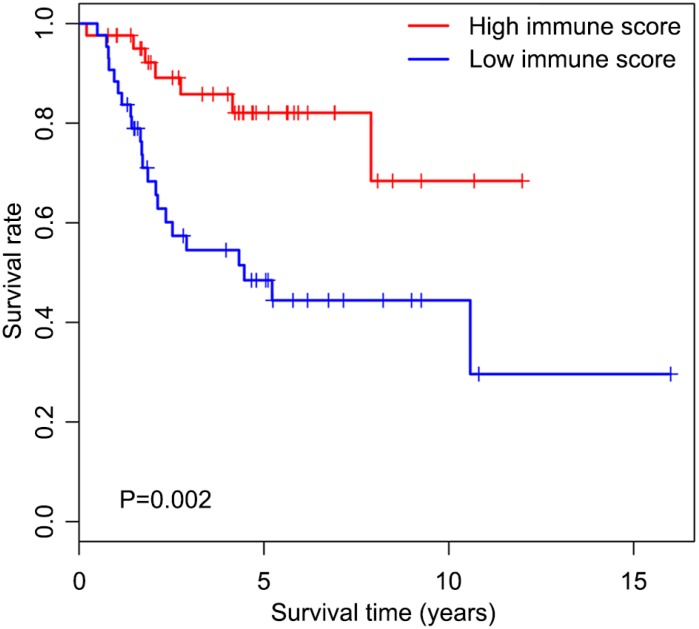
**Overall survival curves obtained by the Kaplan-Meier method indicate that the immune score is significantly associated with OS prognosis.** Horizontal and vertical axes represent survival times and survival rates, respectively. Red and blue curves are samples with immune score higher and lower than the median value, respectively. Plus signs are censored values. Depicted P-values were obtained by the log rank test. OS, osteosarcoma.

We first determined immune score of the normalized matrix data of 85 OS samples with complete clinical data by applying the ESTIMATE algorithm. Subsequently, the OS cases were assigned to the high and low immune score groups respectively, according to the median value of immune scores. Kaplan-Meier curves revealed that the high immune score was significantly associated with improved outcome (P=0.002) (Figure 1). The five-year survival rates in cases with high and low immune score were 82.1% and 48.5%, respectively.

### Compositions of TIICs in OS patients with high and low immune score

**Figure 2:**
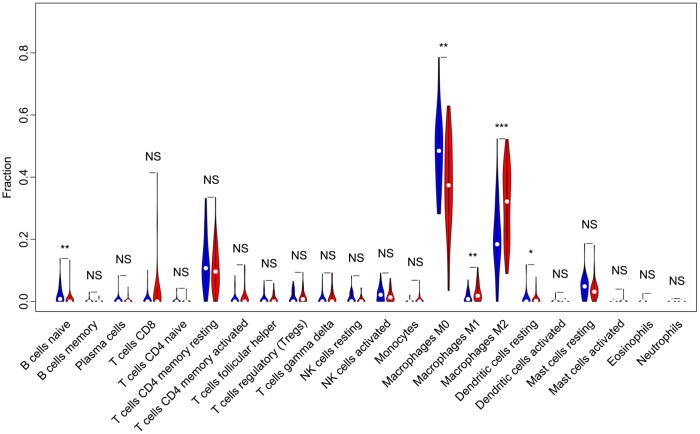
**Violin plot comparing the proportions of TIICs between low and high immune score OS samples.** Horizontal and vertical axes respectively represent TIICs and relative percentages. Blue and red colors represent low and high immune score OS samples, respectively. Data were assessed by the Wilcoxon rank-sum test. ^*^P<0.05, ^**^P<0.01, ^***^P<0.001. NS, no significance; TIICs, tumor-infiltrating immune cells; OS, osteosarcoma; NK, natural killer.

**Figure 3:**
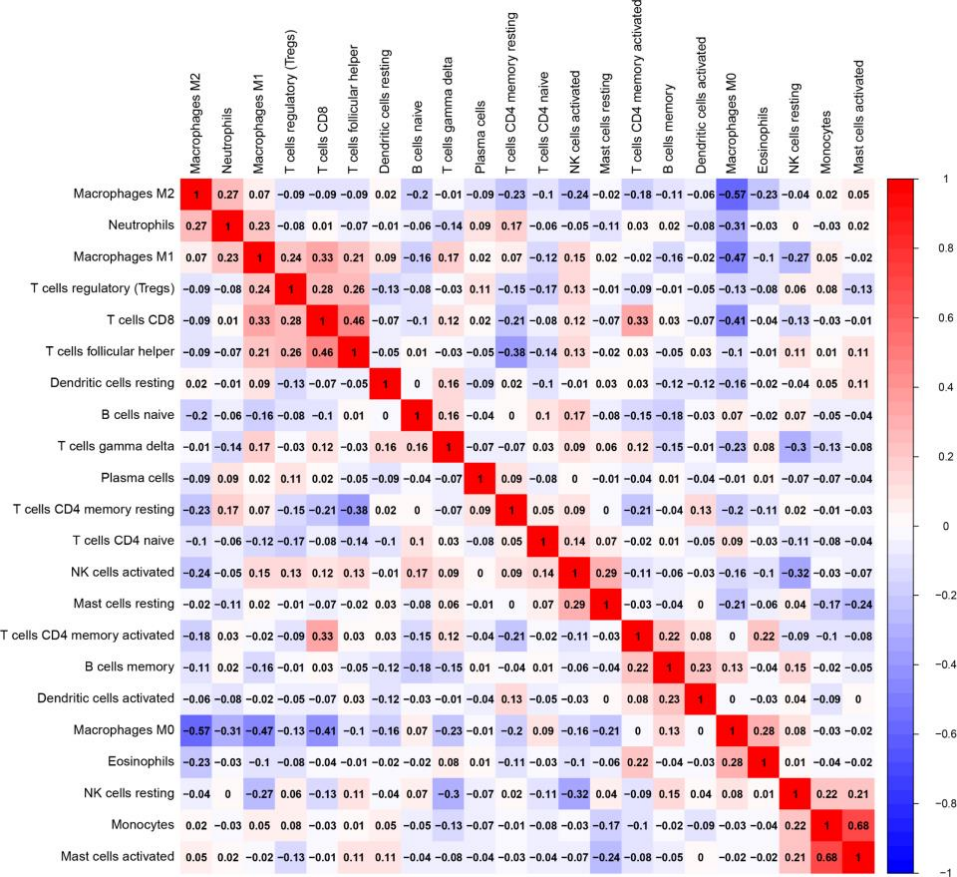
**Correlation matrix of all 22 TIICs proportions.** Horizontal and vertical axes both represent TIICs. TIICs with higher, lower, and same correlation levels are shown in red, blue, and white, respectively. TIIC, tumor-infiltrating immune cell.

Of all OS samples, 38 and 43 with low and high immune score, respectively, were eligible based on CIBERSORT P<0.05. The two most common TIICs in OS tissues were macrophages and T lymphocytes, which accounted for more than 80% of all TIICs. Specifically, the proportions of naive B cells (Z=-3.014, P=0.003) and M0 macrophages (Z=-3.095, P=0.002) were significantly lower in high immune score tissues compared with the low immune score group, while the proportions of M1 macrophages (Z=-3.047, P=0.002), M2 macrophages (Z=-3.785, P<0.001) and resting dendritic cells (Z=-2.251, P=0.024) were significantly higher (Figure 2). Furthermore, the ratio of M1 macrophages to total polarized macrophages (M1 and M2) showed no significant difference between high and low immune score tissues (Z=-1.427, P=0.154). Correlations among the 22 TIICs ranged from weak to moderate. Obviously, M0 macrophages showed highly negative correlations with M1 and M2 macrophages (Figure 3).

### Gene expression profiles in high and low immune score OS tissues

**Figure 4:**
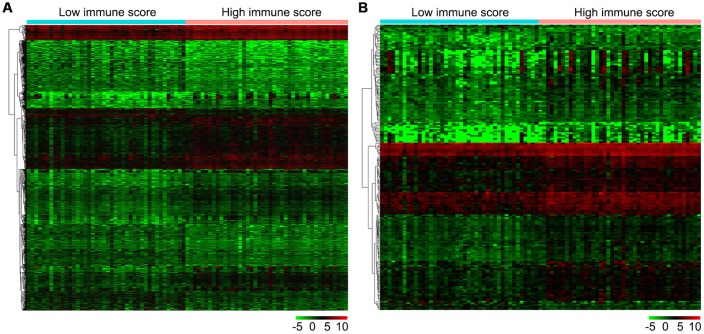
**Gene expression profiles in high and low immune score OS samples.** (**A**) Heat map of DEGs based on immune score in OS samples. (**B**) Heat map of immune-related DEGs based on immune score in OS samples. Horizontal and vertical axes represent OS samples and genes, respectively. Genes with higher, lower, and same expression levels are shown in red, green, and black, respectively. Color bars on top of the heat map represent sample types, with blue and pink indicating low and high immune score samples, respectively. DEGs, differentially expressed genes; OS, osteosarcoma.

Firstly, we compared 42 low and 43 high immune score OS samples of the normalized matrix data. Compared with the low immune score group, there were 607 upregulated and 459 downregulated DEGs in the high immune score group (Figure 4A). Subsequently, we identified immune-related DEGs. Compared with the low immune score group, there were 177 upregulated DEGs and 14 downregulated immune-related DEGs in high immune score specimens (Figure 4B).

### Correlation of the immune-related risk score with overall survival

**Figure 5:**
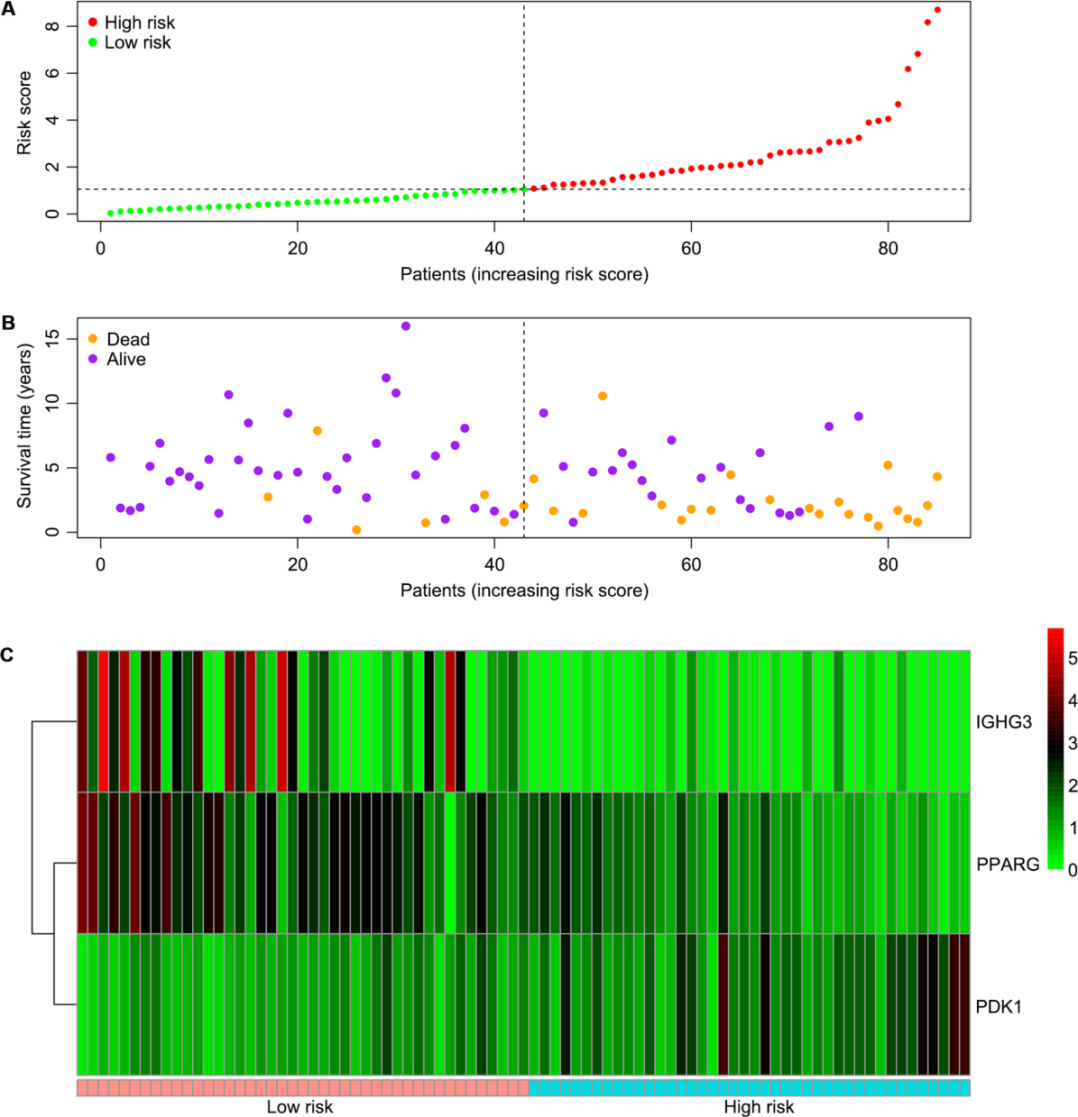
**Associations of the risk score with the expression levels of three immune-related genes included in the risk model.** (**A**) Dot plot of risk score. Vertical and horizontal axes respectively represent risk score and OS samples, ranked by increasing risk score. Red and green colors represent high and low risk cases, respectively. (**B**) Dot plot of survival. Vertical and horizontal axes respectively represent survival times and OS samples, ranked by increasing risk score. Orange and purple colors represent dead and living OS cases, respectively. (**C**) Heat map of the expression levels of the three genes. Vertical and horizontal axes respectively represent genes and OS samples, ranked by increasing risk score. Genes with higher, lower, and same expression levels are shown in red, green, and black, respectively. Color bars at the bottom of the heat map represent sample types, with pink and blue indicating low and high risk score samples, respectively. OS, osteosarcoma.

**Figure 6:**
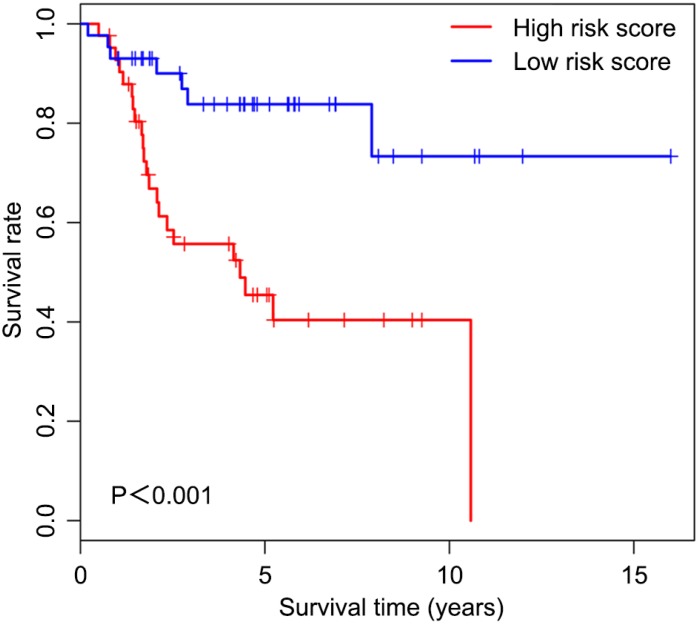
**Overall survival curves obtained by the Kaplan-Meier method indicate that the risk score is significantly associated with OS prognosis.** Horizontal and vertical axes represent survival times and rates, respectively. Red and blue curves are samples with risk score higher and lower than the median value, respectively. Plus signs indicate censored values. Depicted P-values were obtained by the logrank test. OS, osteosarcoma.

**Figure 7:**
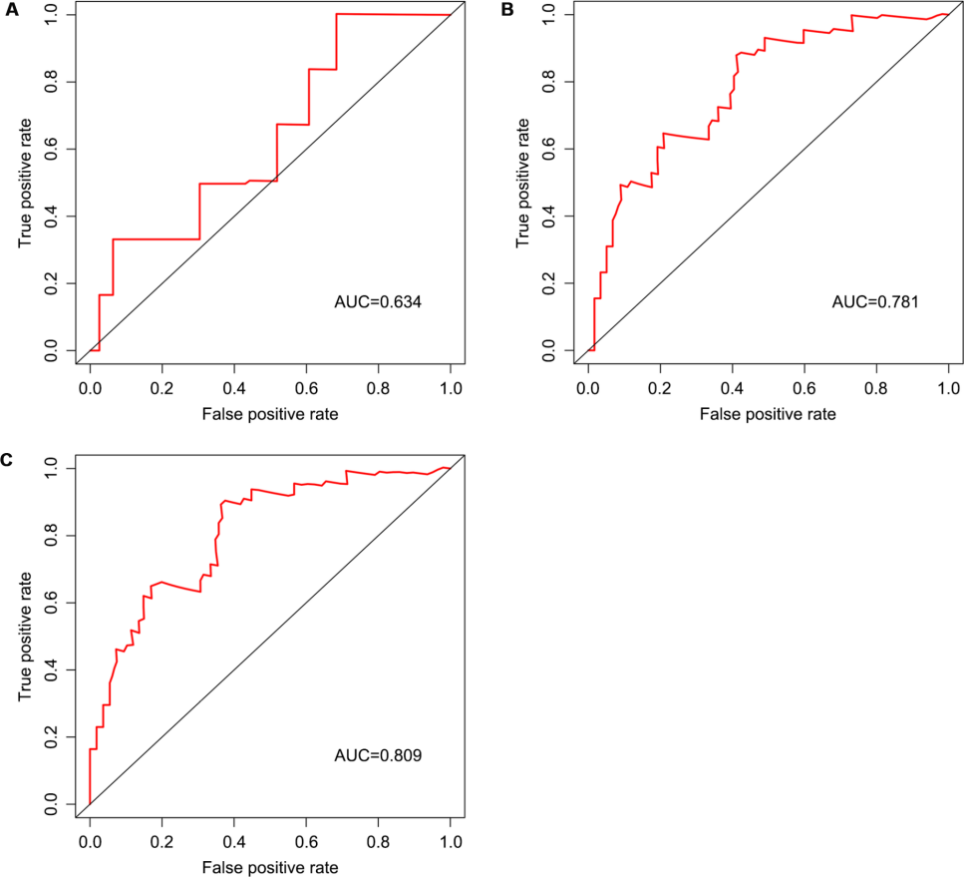
**Survival prediction based on the risk score, determined by time-dependent ROC curve.** Horizontal and vertical axes are false positive and true positive rates, respectively. The AUC values for the risk model in predicting the 1-year (**A**) 3-year (**B**) and 5-year (**C**) survival were 0.634, 0.781, and 0.809, respectively. ROC, receiver operating characteristic; AUC, area under the curve.

Univariable Cox regression analysis revealed 34 immune-related genes which were significantly associated with improved outcome (P<0.05) (Table 1). To assess multicollinearity among different covariates in the model, we excluded variables with variance inflation factor (VIF) >5 (Table 2). A total of 15 genes were excluded, while 19 were included in multivariate Cox regression analysis. Finally, a minimum Akaike information criterion (AIC) value of 210.64 was estimated by the R software to develop the optimal multivariate Cox regression model. The predictive model was then built with three genes, including peroxisome proliferator activated receptor gamma (PPARG), immunoglobulin heavy constant gamma 3 (IGHG3), and pyruvate dehydrogenase kinase 1 (PDK1). Based on relative coefficients in multivariable Cox regression analysis, the following formula was obtained for the risk score: (-0.7728 * PPARG expression level) + (-0.3620 * IGHG3 expression level) + (0.4210 * PDK1 expression level). The median value of risk scores was used as a cutoff to divide samples into two groups (Figure 5A). As shown in Figure 5B, the number of deaths was significantly higher while overall survival was shorter in high-risk cases compared with the low-risk group. Furthermore, in comparison with the low-risk group, high-risk cases had lower expression levels of IGHG3 and PPARG, and higher PDK1 amounts (Figure 5C). High risk score was significantly associated with poor outcome (P<0.01), revealing this score as a good predictive tool (Figure 6). The five-year survival rates of high and low risk score cases were 45.4% and 83.8%, respectively. Moreover, the areas under the curve (AUC) for the risk model in predicting 1, 3 and 5-year survival were 0.634, 0.781, and 0.809, respectively (Figure 7A–7C).

**Table 1:** Univariate and multivariate regression analyses of prognostic factors for overall survival.

**Variables**	**Categories**	**Univariate COX analysis**		**Multivariate COX analysis**	
		**HR(95% CI)**	**P-value**	**HR(95% CI)**	**P-value**
PPARG	High/low	0.456(0.287, 0.723)	0.001	0.462(0.281, 0.760)	0.002
IGHG3	High/low	0.619(0.397, 0.965)	0.034	0.696(0.467, 1.038)	0.075
PDK1	High/low	2.115(1.386, 3.227)	0.001	1.523(0.982, 2.363)	0.060
CD209	High/low	0.575(0.366, 0.902)	0.016		
CCL8	High/low	0.519(0.299, 0.900)	0.020		
TLR2	High/low	0.507(0.295, 0.871)	0.014		
TLR7	High/low	0.459(0.221, 0.951)	0.036		
TLR8	High/low	0.221(0.052, 0.944)	0.042		
GRN	High/low	0.589(0.393, 0.882)	0.010		
TLR1	High/low	0.463(0.214, 0.999)	0.050		
MSR1	High/low	0.635(0.418, 0.965)	0.033		
SLC11A1	High/low	0.463(0.221, 0.970)	0.041		
CD14	High/low	0.774(0.601, 0.997)	0.048		
HMOX1	High/low	0.736(0.556, 0.974)	0.032		
CCL2	High/low	0.580(0.389, 0.866)	0.008		
IL10	High/low	0.186(0.041, 0.845)	0.029		
FCER1G	High/low	0.719(0.555, 0.931)	0.012		
HCK	High/low	0.656(0.436, 0.988)	0.044		
VAV1	High/low	0.470(0.257, 0.859)	0.014		
CARD11	High/low	0.349(0.133, 0.915)	0.032		
PIK3R5	High/low	0.318(0.135, 0.749)	0.009		
LILRB3	High/low	0.295(0.100, 0.875)	0.028		
FCGR2B	High/low	0.315(0.121, 0.822)	0.018		
IGHG2	High/low	0.675(0.471, 0.968)	0.032		
IGLC2	High/low	0.728(0.542, 0.978)	0.035		
FPR1	High/low	0.450(0.249, 0.812)	0.008		
TNFSF8	High/low	0.223(0.079, 0.625)	0.004		
C3AR1	High/low	0.610(0.419, 0.888)	0.010		
CSF3R	High/low	0.360(0.152, 0.856)	0.021		
IL2RA	High/low	0.215(0.068, 0.679)	0.009		
IL2RG	High/low	0.617(0.392, 0.973)	0.038		
LCP2	High/low	0.534(0.307, 0.929)	0.026		
PRF1	High/low	0.544(0.296, 0.998)	0.049		
PTPRC	High/low	0.621(0.392, 0.985)	0.043		

**Table 2:** Variable filtration by multicollinearity diagnostics.

**Variables**	**Unstandardized coefficients**		**Standardized coefficients**	**t**	**Sig.**	**Collinearity statistics**	
	**B**	**Std. Error**	**Beta**			**Tolerance**	**VIF**
(Constant)	2.116	1.894		1.117	0.268		
CD209	-0.494	0.689	-0.158	-0.718	0.475	0.255	3.916
TLR2	-0.189	0.820	-0.046	-0.230	0.819	0.314	3.182
TLR1	0.722	1.165	0.121	0.619	0.538	0.323	3.093
SLC11A1	0.391	1.193	0.077	0.328	0.744	0.224	4.460
HMOX1	0.342	0.335	0.169	1.020	0.311	0.450	2.224
CCL2	0.448	0.469	0.182	0.953	0.344	0.336	2.980
IL10	-0.247	1.559	-0.030	-0.158	0.875	0.352	2.844
PPARG	1.219	0.487	0.365	2.505	0.015	0.579	1.727
HCK	-0.039	0.777	-0.011	-0.050	0.960	0.250	4.007
LILRB3	-2.311	1.651	-0.325	-1.400	0.166	0.227	4.396
FCGR2B	0.336	1.065	0.059	0.316	0.753	0.350	2.859
IGHG3	-0.124	0.300	-0.058	-0.414	0.680	0.621	1.611
FPR1	0.351	0.810	0.093	0.433	0.666	0.267	3.742
CSF3R	-1.273	1.297	-0.209	-0.982	0.330	0.271	3.684
IL2RA	-0.403	1.125	-0.067	-0.358	0.721	0.346	2.890
IL2RG	0.610	0.608	0.198	1.002	0.320	0.315	3.175
PRF1	-0.172	0.691	-0.047	-0.248	0.805	0.349	2.868
PTPRC	-0.330	0.837	-0.091	-0.394	0.695	0.233	4.291
PDK1	-0.362	0.602	-0.084	-0.601	0.550	0.635	1.575

### Correlation of the immune-related risk score with the proportions of TIICs

As shown in Table 3, the risk score was positively correlated with the proportions of memory B cells (r=0.252, P<0.05), M0 macrophages (r=0.305, P<0.01) and resting dendritic cells (r=0.246, P<0.05), and negatively correlated with those of gamma delta T cells (r=-0.245, P<0.05) and M2 macrophages (r=-0.244, P<0.05).

**Table 3:** Spearman rank analysis to determine the association between risk score and the levels of 22 TIICs in OS tissues.

**Tumor-infiltrating immune cell**	**Risk score**	
	**Correlation coefficient**	**P-value**
B cells memory	0.252	0.023
B cells naive	0.090	0.425
Dendritic cells activated	0.093	0.408
Dendritic cells resting	0.246	0.027
Eosinophils	-0.062	0.580
Macrophages M0	0.305	0.006
Macrophages M1	-0.215	0.054
Macrophages M2	-0.244	0.028
Mast cells activated	-0.028	0.805
Mast cells resting	0.010	0.927
Monocytes	-0.053	0.638
Neutrophils	-0.097	0.390
NK cells activated	-0.041	0.715
NK cells resting	0.130	0.248
Plasma cells	-0.129	0.251
T cells CD4 memory activated	-0.149	0.185
T cells CD4 memory resting	0.057	0.612
T cells CD4 naive	-0.010	0.927
T cells CD8	-0.153	0.172
T cells follicular helper	-0.004	0.968
T cells gamma delta	-0.245	0.027
T cells regulatory (Tregs)	-0.086	0.448

### The immune related risk score predicts the involvement of immune pathways

**Figure 8:**
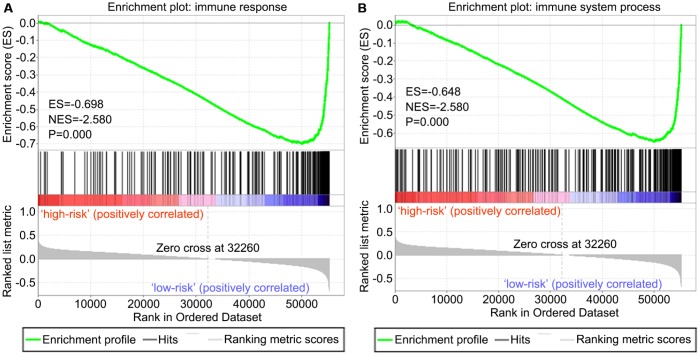
**GSEA of the risk score in OS. Both immune response and immune system process gene sets were enriched in the low-risk group.** The horizontal axis represents genes of the immune response (**A**) and immune system process (**B**) gene sets, ranked by decreasing risk score. The vertical axis represents enrichment score. The enrichment score increased with the number of enriched genes and vice versa. ES, enrichment score; NES, normalized enrichment score.

Two immune gene sets, including M19817 (immune response) and M13664 (immune system process), were retrieved from the Molecular Signatures Database v4.0 (http://software.broadinstitute.org/gsea/downloads.jsp). As shown in Figure 8A, 8B by gene set enrichment analysis (GSEA), both immune response and immune system process gene sets were significantly enriched in the low-risk group (P<0.001).

## DISCUSSION

As is calculated by the ESTIMATE algorithm, it is well admitted that elevated immune score is significantly correlated with poor prognosis in clear cell renal cell cancer patients [13]. This aroused our interest in exploring a potential association of immune score with survival in OS patients. To the best of our knowledge, this is the first study building an immune-related risk model to predict outcome in patients with OS by mining the TCGA database. In the present study, we firstly evaluated approximate proportions of TIICs in OS TME, calculating immune score by applying the ESTIMATE algorithm. Importantly, a high correlation was found between the immune score and overall survival in OS patients. It has been demonstrated that TIICs are significantly relevant to the progression and prognosis of OS [18]. In order to explore specific differences in the proportions of TIICs, OS cases were assigned to the high and low immune score groups. Then, the types of TIICs were assessed in both groups of OS tissues with CIBERSOTR. Subsequently, immune-related DEGs were screened between high and low immune score OS tissues, and an optimal immune-related risk model was built by univariate and multivariate Cox regression analyses. In this model, high risk score, calculated by the expression levels of three immune-related DEGs, was associated with poor outcome. Moreover, of these three genes, PDK1 overlapped with the gene signatures found on the CIBERSORT platform, which implied that immune-related risk score and the proportions of TIICs may be somehow associated. Fortunately, correlations of the risk score with the proportions of 5 TIICs were determined in this study. Finally, we applied GSEA to assess the associations of immune pathways with the determined risk score.

It has been reported that tumor-associated macrophages (TAMs) and T-lymphocytes are the main components of the immune environment in OS [19], in agreement with the above results. We found that OS cases with elevated immune cell infiltration in the microenvironment had better prognosis. Compared with low immune score cases, patients with high immune score showed markedly decreased levels of M0 macrophages and significantly increased amounts of M1 and M2 macrophages, especially M2 macrophages. Furthermore, the risk score was negatively correlated with the proportions of M1 and M2 macrophages, and positively correlated with the proportion of M0 macrophages, suggesting that the polarization level of M0 to M1 or M2 macrophages may be associated with improved outcome in OS patients. In preclinical models of OS, M2-TAMs are associated with increased tumor growth, metastatic dissemination and vascularization [18]. Excitingly, contrary to findings reported for other solid tumors, such as gastric cancer [15], lung adenocarcinoma [20], and colon cancer [21], studies by Anne Gomez-Brouchet et al. [22] indicated that the presence of CD163-positive M2-polarized macrophages is essential for inhibiting OS progression, which represents an important discovery. However, Buddingh et al. [23] described TAMs in OS as a heterogeneous cell population with both M2 pro-tumor and M1 anti-tumor characteristics. Interestingly, Cristiana Guiducci et al. [24] reported the plasticity of TAMs, with CpG combined with anti-interleukin-10 receptor antibodies readily switching them from M2 to M1. Recently, switching TAMs from M2 to M1 has been suggested for developing novel treatments [25]. It has been demonstrated that all-trans retinoic acid suppresses pulmonary metastasis of OS cells by inhibiting M2-like TAMs [26], which may lead to clinical application in metastatic OS. Based on the above findings, we further analyzed the balance between M1 and M2 macrophages in OS tissues. The ratio of M1 macrophages to total polarized macrophages (M1 and M2) was only slightly elevated in the high immune score group (6.040%) compared with the low immune score group (4.741%), but this difference was not statistically significant. Therefore, we speculated that small changes in the balance of polarized macrophages may be an important factor affecting the prognosis of OS patients.

In this study, the proportion of resting dendritic cells in high immune score tissues was significantly higher than that of the low score group. Moreover, the risk score was positively correlated with the proportion of resting dendritic cells, implying that the activation level of dendritic cells may be associated with improved outcome in OS patients. Masanori Kawano et al. [27] reported that combining agonist anti-glucocorticoid-induced tumor necrosis factor receptor (GITR) antibodies with tumor lysate-pulsed dendritic cells reduces the amounts of immunosuppressive cytokines in OS tissues as well as serum. Furthermore, it has been confirmed that pulsing of dendritic cells with LM8 cell lysate, derived from OS, efficiently enhances CD4^+^ and CD8^+^ T cell proliferation and decreases serum interleukin-4 [28]. While assessing synergistic effects with chemotherapy, it was found that combining doxorubicin, which induces immunogenic cell death, with resting dendritic cells boosts systemic immune reactions, leading to OS inhibition in mouse models [29]. Currently, the dendritic cell-based vaccine, a form of active specific immunotherapy, is considered to confer a possible overall survival advantage in children with cancer, similar to findings in adults [30]. The most promising clinical effects were observed in cases with limited disease or complete response, in whom the complete response state could be maintained upon dendritic cell-vaccination, preventing the tumor from recurring. Conversely, dendritic cell-vaccination shows reduced effects in cases with progressive disease or elevated residual tumor load, most likely for the extremely high immunosuppressive burden of malignant cells, with insufficient time to produce appropriate antitumor immune reactions [31].

We screened differentially expressed immune-associated DEGs, and performed univariable and multivariable Cox analyses to generate a risk model for predicting the prognosis of OS patients. Three DEGs were used to construct the model. PPARG and IGHG3 are two protective immune-related DEGs, while PDK1 is a risk immune-related DEG. The AUC values for the risk model in predicting 1, 3 and 5-year survival were 0.634, 0.781, and 0.809, which indicates a good capability for predicting survival in OS patients of the three-gene combination. Cases were assigned to the high- and low-risk groups based on the median risk score. More than 40% of high-risk cases died within three years of diagnosis, while less than 14% died in the low-risk group. Therefore, extremely frequent follow-up and more aggressive treatments should be applied in the high-risk group. Finally, GSEA further confirmed the close connection of the risk signature with immune pathways. As shown above, immune response and immune system process gene sets were significantly enriched in the low-risk group, which suggests that immunosuppression may exist in high-risk OS patients, and is associated with poor outcome.

PPARG is a ligand-activated transcription factor, belonging to the nuclear hormone receptor family [32]. Accumulating evidence confirms that activation of PPARG could confer inhibitory effects on tumors. A meta-analysis identified an association of PPARG c.1347 C > T polymorphism with elevated risk of developing malignancies such as glioblastoma and esophageal cancer [33]. In clear cell renal cell cancer, PPARG suppresses cell migration and proliferation and induces apoptosis by inhibiting SIX homeobox 2 [34]. Sabatino et al. [35] reported that ring finger domains 1 regulates PPARG negatively and is associated with higher clonogenic, proliferative and migratory potential in colorectal cancer. These findings suggest that PPARG might also represent a tumor suppressor gene in OS. In addition, it has been demonstrated that PPARG is a direct target of miR-27a by luciferase reporter assays [36]. Analysis of differentially expressed miRNAs and their target genes in OS samples showed that miR-324-5p targets PPARG [37], which needs to be verified in future experiments. Furthermore, PPARG expression is independently associated with prolonged survival in colorectal cancer, as demonstrated in two separate prospective cohorts [38], corroborating the findings of the present study in OS patients.

IGHG3 is a member of the immunoglobulin G family [39]. Several studies indicated that IGHG3 is overexpressed in multiple cancer types, such as prostate, breast, and lung cancers, which can differentiate tumor from normal tissues [40–42]. Hsu et al. [39] suggested that IGHG3 expression is tightly associated with improved outcome in breast cancer, which is consistent with our findings in OS. The relationship between IGHG3 expression and OS progression deserves further attention.

PDK1 is a hypoxia-inducible factor-1α target antagonizing pyruvate dehydrogenase (PDH), a pivotal rate-limiting enzyme of the tricarboxylic acid cycle. In hypoxia, pyruvate transformation into acetyl-CoA is suppressed due to PDK1-dependent inhibition of PDH, reducing the amounts of glucose-derived pyruvate entering the tricarboxylic acid cycle [43, 44]. It was reported that PDK1 downregulation in metastatic breast cancer greatly alters the tumor cell capability to utilize glucose as an energy source for the mitochondria under hypoxic or limited glucose conditions. Moreover, for coping mechanisms against stress during metastasis, PDK1 mediates the adaptability of breast cancer cells metastasizing to the liver [45]. Meanwhile, Liu et al. [46] confirmed that downregulation of PDK1 could inhibit migration and metastasis in human breast cancer cells. In addition, overexpression of PDK1 has been reported in multiple myeloma [47], acute myeloid leukemia [48], breast cancer [49] and OS [50]. Li et al. [50] demonstrated that overexpression of PDK1 promotes the proliferation of OS cells. More importantly, PDK1 is a direct target of miR-379, which functions as a tumor-inhibiting miRNA by targeting PDK1 in OS. Two novel PDK1 inhibitors were shown to concentration-dependently reduce the phosphorylation of the pyruvate dehydrogenase complex in MG-63 OS cells, whose proliferation was inhibited as a result [51].

There were several limitations in this study. First, the number of OS tissue samples in the TCGA cohort was relatively small, which could lead to some bias. Secondly, the landscape differences of TIICs and immune-related DEGs between tumor and normal samples were not analyzed in the TCGA cohort because noncancerous samples were not included, and sampling normal bone tissue is subject to restriction in clinic to some extent. Thus, the present findings could only be applied to predict the prognosis of OS patients with definite diagnosis. Thirdly, because the clinical information in the database does not include the tumor stages of OS tissues, we could not perform subgroup analysis based on tumor stage. However, gene expression in OS tissues of different tumor stages may be different, and further research is warranted to address this issue. Finally, the present study performed no external validation based on other available databases; therefore, the current conclusion requires validation in future experiments.

In summary, according to the ESTIMATE algorithm-based immune score that was significantly correlated with improved outcome, 22 TIICs in OS TME were assessed for their levels. Then, a list of immune-related DEGs was extracted, and three such genes (PPARG, IGHG3, and PDK1) were included in a predictive risk model, which could assist clinicians in assessing the prognosis of OS patients and selecting appropriate targets for immunotherapy.

## MATERIALS AND METHODS

### Data collection and processing

The gene expression quantification data of 88 OS samples were of the HTSeq-FPKM type, and downloaded from TCGA (version 18.0) on August 17, 2019. Updated clinical data related to these OS samples, such as age, gender, race, overall survival time and vital status, were also downloaded from the TARGET database (https://ocg.cancer.gov/programs/target) on August 17, 2019. In order to screen out the matrix data of mRNAs with gene properties, the gene expression profiles were compared with the human genome annotation GTF file, which was downloaded from the GenCode platform (https://www.gencodegenes.org/). Subsequently, the matrix data of the gene expression values was organized by the Perl software (version 5.24.3) (https://www.perl.org/).

### Immune score determination for the OS microenvironment

The matrix data of gene expression amounts were normalized with the limma package of the R software (version 3.5.2) [52]. Then, immune score was calculated by applying the ESTIMATE algorithm to the matrix data [11]. Furthermore, the OS cases were assigned to high- and low immune score groups based on the median value of immune scores, to identify a possible association of immune score with overall survival.

### Analysis of the relative proportions of TIICs in OS tissues

TIICs in OS samples from the TCGA cohort were assessed by applying the CIBERSORT deconvolution algorithm. The gene expression signature matrix of 22 TIICs was obtained from the CIBERSORT platform (https://cibersortx.stanford.edu/). The matrix data of gene expression levels were compared with those of the signature matrix of 22 TIICs from the CIBERSORT platform to generate a proportion matrix for the 22 TIICs in OS tissues of the high and low immune score groups using support vector regression [53]. By Monte Carlo sampling, the algorithm derives a P-value for the deconvolution of each sample, offering a measure of confidence for the obtained data. The results of the inferred proportions of TIICs assessed by CIBERSORT were considered to be accurate at a threshold of P<0.05 [17]. Therefore, only samples with a CIBERSORT P<0.05 were deemed qualified for further analysis. Moreover, the number of permutations of the default signature matrix was set to 100.

### Analysis of immune-related DEGs in OS tissues

To further examine the DEGs between low and high immune score OS samples of the TCGA cohort, the normalized matrix data of gene expression levels were analyzed with the limma package of the R software. Log fold change >1 or <-1 and adjusted P<0.05 were set as cut-offs for filtering DEGs. In addition, immune-associated genes were retrieved from the ImmPort platform (https://www.immport.org/) [54], and used to identify immune-related DEGs for constructing predictive risk model.

### Statistical analysis

Only samples with complete clinical data were included in survival analysis, and the logrank test was performed for comparing Kaplan-Meier curves between groups. Differences and correlations among TIICs were analyzed with the vioplot (https://cran.r-project.org/web/packages/vioplot/index.html) and corrplot (https://cran.r-project.org/web/packages/corrplot/index.html) packages of the R software. The differential proportions of the 22 TIICs in the TCGA cohort were evaluated by the Wilcoxon rank-sum test. A heat map was generated using the pheatmap package of the R software (https://cran.r-project.org/web/packages/pheatmap/index.html). The Cox proportional-hazards model was used for analyzing associations of the levels of immune-related DEGs with overall survival. Collinearity diagnostics was performed with the SPSS software (version 24.0) (SPSS, USA). The multicollinearity of each variable was estimated by calculating the VIF. A variable with VIF>5 was considered to show high collinearity [55], and would be excluded from multivariable Cox regression analysis. The optimal multivariable Cox regression model was selected according to the lowest AIC [56]. Coefficients for each covariate were determined by the multivariable Cox regression model, and a total risk score was calculated. The specificity and sensitivity of survival prediction according to the determined risk score were obtained by time-dependent receiver operating characteristic (ROC) curves, with AUC values quantified with the survivalROC package (https://cran.r-project.org/web/packages/survivalROC/index.html). Next, the association of the immune-related risk score with TIIC levels was assessed by spearman rank correlation using the R software. GSEA (http://www.broadinstitute.org/gsea/index.jsp) was carried out to evaluate associations of immune pathways with the immune-related risk score using the GSEA software (version 4.0.1) [57]. P< 0.05 indicated statistical significance.

## References

[1] Moriarity BS, Otto GM, Rahrmann EP, Rathe SK, Wolf NK, Weg MT, Manlove LA, LaRue RS, Temiz NA, Molyneux SD, Choi K, Holly KJ, Sarver AL, et al. A Sleeping Beauty forward genetic screen identifies new genes and pathways driving osteosarcoma development and metastasis. Nat Genet. 2015; 47:615–24. 10.1038/ng.3293 25961939 PMC4767150

[2] Nie Z, Peng H. Osteosarcoma in patients below 25 years of age: an observational study of incidence, metastasis, treatment and outcomes. Oncol Lett. 2018; 16:6502–14. 10.3892/ol.2018.9453 30405789 PMC6202522

[3] Siegel RL, Miller KD, Jemal A. Cancer statistics, 2018. CA Cancer J Clin. 2018; 68:7–30. 10.3322/caac.21442 29313949

[4] Isakoff MS, Bielack SS, Meltzer P, Gorlick R. Osteosarcoma: Current Treatment and a Collaborative Pathway to Success. J Clin Oncol. 2015; 33:3029–35. 10.1200/JCO.2014.59.4895 26304877 PMC4979196

[5] Liu Y, Qiao L, Zhang S, Wan G, Chen B, Zhou P, Zhang N, Wang Y. Dual pH-responsive multifunctional nanoparticles for targeted treatment of breast cancer by combining immunotherapy and chemotherapy. Acta Biomater. 2018; 66:310–24. 10.1016/j.actbio.2017.11.010 29129789

[6] Guerra AD, Yeung OW, Qi X, Kao WJ, Man K. The Anti-Tumor Effects of M1 Macrophage-Loaded Poly (ethylene glycol) and Gelatin-Based Hydrogels on Hepatocellular Carcinoma. Theranostics. 2017; 7:3732–44. 10.7150/thno.20251 29109772 PMC5667344

[7] Hanahan D, Coussens LM. Accessories to the crime: functions of cells recruited to the tumor microenvironment. Cancer Cell. 2012; 21:309–22. 10.1016/j.ccr.2012.02.022 22439926

[8] Cortini M, Avnet S, Baldini N. Mesenchymal stroma: role in osteosarcoma progression. Cancer Lett. 2017; 405:90–99. 10.1016/j.canlet.2017.07.024 28774797

[9] Wang C, Zhou X, Li W, Li M, Tu T, Ba X, Wu Y, Huang Z, Fan G, Zhou G, Wu S, Zhao J, Zhang J, Chen J. Macrophage migration inhibitory factor promotes osteosarcoma growth and lung metastasis through activating the RAS/MAPK pathway. Cancer Lett. 2017; 403:271–79. 10.1016/j.canlet.2017.06.011 28642171

[10] Koirala P, Roth ME, Gill J, Piperdi S, Chinai JM, Geller DS, Hoang BH, Park A, Fremed MA, Zang X, Gorlick R. Immune infiltration and PD-L1 expression in the tumor microenvironment are prognostic in osteosarcoma. Sci Rep. 2016; 6:30093. 10.1038/srep30093 27456063 PMC4960483

[11] Yoshihara K, Shahmoradgoli M, Martínez E, Vegesna R, Kim H, Torres-Garcia W, Treviño V, Shen H, Laird PW, Levine DA, Carter SL, Getz G, Stemke-Hale K, et al. Inferring tumour purity and stromal and immune cell admixture from expression data. Nat Commun. 2013; 4:2612. 10.1038/ncomms3612 24113773 PMC3826632

[12] Jia D, Li S, Li D, Xue H, Yang D, Liu Y. Mining TCGA database for genes of prognostic value in glioblastoma microenvironment. Aging (Albany NY). 2018; 10:592–605. 10.18632/aging.101415 29676997 PMC5940130

[13] Xu WH, Xu Y, Wang J, Wan FN, Wang HK, Cao DL, Shi GH, Qu YY, Zhang HL, Ye DW. Prognostic value and immune infiltration of novel signatures in clear cell renal cell carcinoma microenvironment. Aging (Albany NY). 2019; 11:6999–7020. 10.18632/aging.102233 31493764 PMC6756904

[14] Chen B, Khodadoust MS, Liu CL, Newman AM, Alizadeh AA. Profiling Tumor Infiltrating Immune Cells with CIBERSORT. Methods Mol Biol. 2018; 1711:243–59. 10.1007/978-1-4939-7493-1_12 29344893 PMC5895181

[15] Zeng D, Zhou R, Yu Y, Luo Y, Zhang J, Sun H, Bin J, Liao Y, Rao J, Zhang Y, Liao W. Gene expression profiles for a prognostic immunoscore in gastric cancer. Br J Surg. 2018; 105:1338–48. 10.1002/bjs.10871 29691839 PMC6099214

[16] Xiong Y, Wang K, Zhou H, Peng L, You W, Fu Z. Profiles of immune infiltration in colorectal cancer and their clinical significant: A gene expression-based study. Cancer Med. 2018; 7:4496–508. 10.1002/cam4.1745 30117315 PMC6144159

[17] Ali HR, Chlon L, Pharoah PD, Markowetz F, Caldas C. Patterns of Immune Infiltration in Breast Cancer and Their Clinical Implications: A Gene-Expression-Based Retrospective Study. PLoS Med. 2016; 13:e1002194. 10.1371/journal.pmed.1002194 27959923 PMC5154505

[18] Li X, Chen Y, Liu X, Zhang J, He X, Teng G, Yu D. Tim3/Gal9 interactions between T cells and monocytes result in an immunosuppressive feedback loop that inhibits Th1 responses in osteosarcoma patients. Int Immunopharmacol. 2017; 44:153–59. 10.1016/j.intimp.2017.01.006 28103502

[19] Heymann MF, Lézot F, Heymann D. The contribution of immune infiltrates and the local microenvironment in the pathogenesis of osteosarcoma. Cell Immunol. 2019; 343:103711. 10.1016/j.cellimm.2017.10.011 29117898

[20] Yang X, Shi Y, Li M, Lu T, Xi J, Lin Z, Jiang W, Guo W, Zhan C, Wang Q. Identification and validation of an immune cell infiltrating score predicting survival in patients with lung adenocarcinoma. J Transl Med. 2019; 17:217. 10.1186/s12967-019-1964-6 31286969 PMC6615164

[21] Peng D, Wang L, Li H, Cai C, Tan Y, Xu B, Le H. An immune infiltration signature to predict the overall survival of patients with colon cancer. IUBMB Life. 2019; 71:1760–70. 10.1002/iub.2124 31301220

[22] Gomez-Brouchet A, Illac C, Gilhodes J, Bouvier C, Aubert S, Guinebretiere JM, Marie B, Larousserie F, Entz-Werlé N, de Pinieux G, Filleron T, Minard V, Minville V, et al. CD163-positive tumor-associated macrophages and CD8-positive cytotoxic lymphocytes are powerful diagnostic markers for the therapeutic stratification of osteosarcoma patients: an immunohistochemical analysis of the biopsies fromthe French OS2006 phase 3 trial. OncoImmunology. 2017; 6:e1331193. 10.1080/2162402X.2017.1331193 28932633 PMC5599091

[23] Buddingh EP, Kuijjer ML, Duim RA, Bürger H, Agelopoulos K, Myklebost O, Serra M, Mertens F, Hogendoorn PC, Lankester AC, Cleton-Jansen AM. Tumor-infiltrating macrophages are associated with metastasis suppression in high-grade osteosarcoma: a rationale for treatment with macrophage activating agents. Clin Cancer Res. 2011; 17:2110–19. 10.1158/1078-0432.CCR-10-2047 21372215

[24] Guiducci C, Vicari AP, Sangaletti S, Trinchieri G, Colombo MP. Redirecting in vivo elicited tumor infiltrating macrophages and dendritic cells towards tumor rejection. Cancer Res. 2005; 65:3437–46. 10.1158/0008-5472.CAN-04-4262 15833879

[25] Zanganeh S, Hutter G, Spitler R, Lenkov O, Mahmoudi M, Shaw A, Pajarinen JS, Nejadnik H, Goodman S, Moseley M, Coussens LM, Daldrup-Link HE. Iron oxide nanoparticles inhibit tumour growth by inducing pro-inflammatory macrophage polarization in tumour tissues. Nat Nanotechnol. 2016; 11:986–94. 10.1038/nnano.2016.168 27668795 PMC5198777

[26] Zhou Q, Xian M, Xiang S, Xiang D, Shao X, Wang J, Cao J, Yang X, Yang B, Ying M, He Q. All-Trans Retinoic Acid Prevents Osteosarcoma Metastasis by Inhibiting M2 Polarization of Tumor-Associated Macrophages. Cancer Immunol Res. 2017; 5:547–59. 10.1158/2326-6066.CIR-16-0259 28515123

[27] Kawano M, Tanaka K, Itonaga I, Iwasaki T, Miyazaki M, Ikeda S, Tsumura H. Dendritic cells combined with anti-GITR antibody produce antitumor effects in osteosarcoma. Oncol Rep. 2015; 34:1995–2001. 10.3892/or.2015.4161 26239052

[28] He YT, Zhang QM, Kou QC, Tang B. *In vitro* generation of cytotoxic T lymphocyte response using dendritic cell immunotherapy in osteosarcoma. Oncol Lett. 2016; 12:1101–06. 10.3892/ol.2016.4714 27446401 PMC4950224

[29] Kawano M, Tanaka K, Itonaga I, Iwasaki T, Miyazaki M, Ikeda S, Tsumura H. Dendritic cells combined with doxorubicin induces immunogenic cell death and exhibits antitumor effects for osteosarcoma. Oncol Lett. 2016; 11:2169–75. 10.3892/ol.2016.4175 26998143 PMC4774596

[30] Anguille S, Smits EL, Lion E, van Tendeloo VF, Berneman ZN. Clinical use of dendritic cells for cancer therapy. Lancet Oncol. 2014; 15:e257–67. 10.1016/S1470-2045(13)70585-0 24872109

[31] de Bruijn S, Anguille S, Verlooy J, Smits EL, van Tendeloo VF, de Laere M, Norga K, Berneman ZN, Lion E. Dendritic Cell-Based and Other Vaccination Strategies for Pediatric Cancer. Cancers (Basel). 2019; 11:11. 10.3390/cancers11091396 31546858 PMC6770385

[32] Lehrke M, Lazar MA. The many faces of PPARgamma. Cell. 2005; 123:993–99. 10.1016/j.cell.2005.11.026 16360030

[33] Ding H, Chen Y, Qiu H, Liu C, Wang Y, Kang M, Tang W. PPARG c.1347C>T polymorphism is associated with cancer susceptibility: from a case-control study to a meta-analysis. Oncotarget. 2017; 8:102277–90. 10.18632/oncotarget.20925 29254243 PMC5731953

[34] Wu Y, Song T, Liu M, He Q, Chen L, Liu Y, Ni D, Liu J, Hu Y, Gu Y, Li Q, Zhou Q, Xie Y. *PPARG* Negatively Modulates *Six2* in Tumor Formation of Clear Cell Renal Cell Carcinoma. DNA Cell Biol. 2019; 38:700–07. 10.1089/dna.2018.4549 31090452

[35] Sabatino L, Fucci A, Pancione M, Carafa V, Nebbioso A, Pistore C, Babbio F, Votino C, Laudanna C, Ceccarelli M, Altucci L, Bonapace IM, Colantuoni V. UHRF1 coordinates peroxisome proliferator activated receptor gamma (PPARG) epigenetic silencing and mediates colorectal cancer progression. Oncogene. 2012; 31:5061–72. 10.1038/onc.2012.3 22286757

[36] Tang KQ, Wang YN, Zan LS, Yang WC. miR-27a controls triacylglycerol synthesis in bovine mammary epithelial cells by targeting peroxisome proliferator-activated receptor gamma. J Dairy Sci. 2017; 100:4102–12. 10.3168/jds.2016-12264 28284697

[37] Ma G, Zhang C, Luo W, Zhao JL, Wang X, Qian Y. Construction of microRNA-messenger networks for human osteosarcoma. J Cell Physiol. 2019; 234:14145–53. 10.1002/jcp.28107 30666640 PMC13483061

[38] Ogino S, Shima K, Baba Y, Nosho K, Irahara N, Kure S, Chen L, Toyoda S, Kirkner GJ, Wang YL, Giovannucci EL, Fuchs CS. Colorectal cancer expression of peroxisome proliferator-activated receptor gamma (PPARG, PPARgamma) is associated with good prognosis. Gastroenterology. 2009; 136:1242–50. 10.1053/j.gastro.2008.12.048 19186181 PMC2663601

[39] Hsu HM, Chu CM, Chang YJ, Yu JC, Chen CT, Jian CE, Lee CY, Chiang YT, Chang CW, Chang YT. Six novel immunoglobulin genes as biomarkers for better prognosis in triple-negative breast cancer by gene co-expression network analysis. Sci Rep. 2019; 9:4484. 10.1038/s41598-019-40826-w 30872752 PMC6418134

[40] Ledet EM, Hu X, Sartor O, Rayford W, Li M, Mandal D. Characterization of germline copy number variation in high-risk African American families with prostate cancer. Prostate. 2013; 73:614–23. 10.1002/pros.22602 23060098

[41] Bin Amer SM, Maqbool Z, Nirmal MS, Qattan AT, Hussain SS, Jeprel HA, Tulbah AM, Malik OA, Al-Tweigeri TA. Gene expression profiling in women with breast cancer in a Saudi population. Saudi Med J. 2008; 29:507–13. 18382789

[42] Remmelink M, Mijatovic T, Gustin A, Mathieu A, Rombaut K, Kiss R, Salmon I, Decaestecker C. Identification by means of cDNA microarray analyses of gene expression modifications in squamous non-small cell lung cancers as compared to normal bronchial epithelial tissue. Int J Oncol. 2005; 26:247–58. 10.3892/ijo.26.1.247 15586247

[43] Kim JW, Tchernyshyov I, Semenza GL, Dang CV. HIF-1-mediated expression of pyruvate dehydrogenase kinase: a metabolic switch required for cellular adaptation to hypoxia. Cell Metab. 2006; 3:177–85. 10.1016/j.cmet.2006.02.002 16517405

[44] Papandreou I, Cairns RA, Fontana L, Lim AL, Denko NC. HIF-1 mediates adaptation to hypoxia by actively downregulating mitochondrial oxygen consumption. Cell Metab. 2006; 3:187–97. 10.1016/j.cmet.2006.01.012 16517406

[45] Dupuy F, Tabariès S, Andrzejewski S, Dong Z, Blagih J, Annis MG, Omeroglu A, Gao D, Leung S, Amir E, Clemons M, Aguilar-Mahecha A, Basik M, et al. PDK1-Dependent Metabolic Reprogramming Dictates Metastatic Potential in Breast Cancer. Cell Metab. 2015; 22:577–89. 10.1016/j.cmet.2015.08.007 26365179

[46] Liu Y, Wang J, Wu M, Wan W, Sun R, Yang D, Sun X, Ma D, Ying G, Zhang N. Down-regulation of 3-phosphoinositide-dependent protein kinase-1 levels inhibits migration and experimental metastasis of human breast cancer cells. Mol Cancer Res. 2009; 7:944–54. 10.1158/1541-7786.MCR-08-0368 19531564

[47] Fujiwara S, Kawano Y, Yuki H, Okuno Y, Nosaka K, Mitsuya H, Hata H. PDK1 inhibition is a novel therapeutic target in multiple myeloma. Br J Cancer. 2013; 108:170–78. 10.1038/bjc.2012.527 23321518 PMC3553526

[48] Zabkiewicz J, Pearn L, Hills RK, Morgan RG, Tonks A, Burnett AK, Darley RL. The PDK1 master kinase is over-expressed in acute myeloid leukemia and promotes PKC-mediated survival of leukemic blasts. Haematologica. 2014; 99:858–64. 10.3324/haematol.2013.096487 24334295 PMC4008098

[49] Arsenic R. Immunohistochemical analysis of PDK1 expression in breast cancer. Diagn Pathol. 2014; 9:82. 10.1186/1746-1596-9-82 24739482 PMC4005628

[50] Li Z, Shen J, Chan MT, Wu WK. MicroRNA-379 suppresses osteosarcoma progression by targeting PDK1. J Cell Mol Med. 2017; 21:315–23. 10.1111/jcmm.12966 27781416 PMC5264134

[51] Fang A, Luo H, Liu L, Fan H, Zhou Y, Yao Y, Zhang Y. Identification of pyruvate dehydrogenase kinase 1 inhibitors with anti-osteosarcoma activity. Bioorg Med Chem Lett. 2017; 27:5450–53. 10.1016/j.bmcl.2017.10.073 29150396

[52] Ritchie ME, Phipson B, Wu D, Hu Y, Law CW, Shi W, Smyth GK. limma powers differential expression analyses for RNA-sequencing and microarray studies. Nucleic Acids Res. 2015; 43:e47. 10.1093/nar/gkv007 25605792 PMC4402510

[53] Newman AM, Liu CL, Green MR, Gentles AJ, Feng W, Xu Y, Hoang CD, Diehn M, Alizadeh AA. Robust enumeration of cell subsets from tissue expression profiles. Nat Methods. 2015; 12:453–57. 10.1038/nmeth.3337 25822800 PMC4739640

[54] Bhattacharya S, Andorf S, Gomes L, Dunn P, Schaefer H, Pontius J, Berger P, Desborough V, Smith T, Campbell J, Thomson E, Monteiro R, Guimaraes P, et al. ImmPort: disseminating data to the public for the future of immunology. Immunol Res. 2014; 58:234–39. 10.1007/s12026-014-8516-1 24791905

[55] Morris JK, Bestwick J, Wald NJ. Multiple-marker screening for Down’s syndrome: a method of assessing the statistical robustness of proposed tests. J Med Screen. 2008; 15:55–61. 10.1258/jms.2008.007105 18573771

[56] Burns RJ, Deschênes SS, Schmitz N. Associations between Depressive Symptoms and Social Support in Adults with Diabetes: Comparing Directionality Hypotheses with a Longitudinal Cohort. Ann Behav Med. 2016; 50:348–57. 10.1007/s12160-015-9760-x 26631086

[57] Subramanian A, Tamayo P, Mootha VK, Mukherjee S, Ebert BL, Gillette MA, Paulovich A, Pomeroy SL, Golub TR, Lander ES, Mesirov JP. Gene set enrichment analysis: a knowledge-based approach for interpreting genome-wide expression profiles. Proc Natl Acad Sci USA. 2005; 102:15545–50. 10.1073/pnas.0506580102 16199517 PMC1239896

